# Clustering crocodylian dental morphology: Insights into functional adaptations, diet, and ontogeny

**DOI:** 10.1111/joa.70014

**Published:** 2025-07-02

**Authors:** Jason J. Testin, Domenic C. D'Amore

**Affiliations:** ^1^ Department of Physics University of Nebraska at Omaha, Durham Science Center Omaha Nebraska USA; ^2^ Department of Physical Science, Physics and Pre‐Engineering Iowa Western Community College Council Bluffs Iowa USA; ^3^ Department of Natural Sciences Daemen University Amherst New York USA

**Keywords:** Alligatoridae, Crocodylidae, ecomorphology, functional adaptations, Gavialidae, heterodonty, teeth

## Abstract

Crocodylians have often been grouped into ecomorphological categories based on snout characters and diet, but quantitative dental morphology has rarely been used for this purpose. We collected Euclidean measurements from the teeth of 18 extant crocodylian species spanning a range of sizes and snout ecomorphotypes, normalized the data for size heterodonty using regression analyses, grouped the crowns into eight dental sections along the arcade, and ran a K‐means cluster analysis to cluster individuals based on shape heterodonty. Five clusters emerged, each reflecting different degrees of gracility or robustness of crowns and their variation along the jaw arcade. These morphological clusters showed a connection to snout shape, prey preference, and feeding ecology, particularly prey size and the degree of processing necessary. Cluster assignments were, for the most part, not taxon specific; multiple families and subfamilies were found in most clusters, and members of the same species were often found in more than one cluster. For species with members in multiple clusters, the larger individuals typically were in the cluster with more robust crowns. This supports prior suggestions that dental morphotype coincides with ontogenetic niche shifts. This approach demonstrates the potential for using dental morphology to infer ecological roles in both extant and fossil crocodylians, paving the way for future comparative analyses of archosaur dentition.

## Graphical abstract

Extant crocodylians may be divided into five clusters based on the residuals of four linear dental measurements (*y*‐axes) and their variation along the jaw (*x*‐axes). These clusters correlate to snout shape, prey preference, and feeding ecology, particularly prey size and the degree of processing necessary. Multiple families and subfamilies were found in most clusters, and members of the same species were often found in more than one cluster as a result of ontogenetic niche shift.

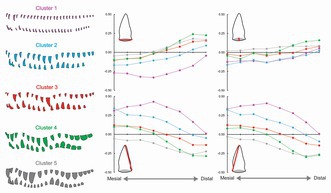

## 1 INTRODUCTION

Dental morphology in amniotes is directly associated with feeding behaviors (Henderson, 1998; Melstrom, 2017). This relationship suggests the potential to correlate dental morphology with such behaviors in extant reptiles. Tooth crowns exhibit a wide range of sizes and shapes to accommodate diverse food sources (Ciampaglio et al., 2005; Massare, 1987; Zahradnicek et al., 2014). Analyzing reptile teeth poses unique challenges due to the relative simplicity of their dentition compared to mammals, as reptiles lack well‐defined dental categories and the multiple cusps that serve as morphological landmarks in mammals (Polly, 2000; Zahradnicek et al., 2014). Additionally, reptile dental morphology often shows overlap among different taxa, making it challenging for taxonomic discrimination (Han et al., 2011). Tooth morphology can also vary significantly within an individual (Westaway et al., 2011). Despite these challenges, studies have successfully examined tooth morphology in numerous reptile taxa both extinct and extant, and have explored many associated facets including heterodonty, taxonomic comparisons, tooth assemblage variation, fossil identification, trophic adaptations, and ecology/paleoecology (Britt et al., 2009; Buckley & Currie, 2014; D'Amore, 2009, 2015; D'Amore et al., 2019, 2024; Foffa et al., 2018, 2024; Gerke & Wings, 2016; Hendrickx et al., 2015; Hendrickx et al., 2020; Hendrickx & Mateus, 2014; Larson et al., 2016; Larson & Currie, 2013; Melstrom & Irmis, 2019; Segall et al., 2023; Smith et al., 2005; Torices et al., 2014). A plethora of techniques have been used in these studies, including Euclidean distances, 2D and 3D landmark‐based morphometrics, and dental complexity analyses.

This study aims to cluster crocodylians based on tooth morphology and associate these clusters with diet and other life history traits. Modern crocodylians have thecodont dentition with monocuspid, conical, non‐denticulate teeth with variable enamel thickness (Brink & Le Blanc, 2023; Sellers et al., 2019). Heterodonty, often referred to as pseudoheterodonty, is present (Kieser et al., 1993; Ristevski, 2019). D'Amore et al. (2019) quantified the shape and size heterodonty of crocodylians, showing a general trend in crown shape from caniniform to molariform. Most crocodilians exhibit size heterodonty with enlarged crowns in the cranial and dentary arcades in a predictable manner (Erickson et al., 2003; Gignac & Erickson, 2015). Fossil crocodylians display broader tooth morphologies, including ziphodont, globidont, and multicupsid representatives (Brochu, 2001; Ristevski et al., 2024; Sobbe et al., 2013). Herbivorous crocodyliforms have evolved numerous times, with these suspected herbivorous taxa generally having more complex crowns compared to carnivores (Melstrom & Irmis, 2019).

Crocodylian cranial morphology also reflects ontogeny, habitats, and diet, emphasizing the importance of feeding adaptations in this group (Busbey, 1995; Cleuren & De Vree, 2000). Crocodylian skulls have been linked to dietary preferences and ecological niches (Brochu, 2001; Pierce et al., 2008; Rhind et al., 2015). Differences in snout shape and tooth morphologies may be related to ecology (Borteiro et al., 2009; Brochu, 2001; Drumheller & Wilberg, 2020). For example, longirostral snouts are associated with higher rates of piscivory, while brevirostral snouts are better suited for a generalist diet. Snout morphology can also correspond to habitat preferences, with long‐snouted taxa favoring riverine habitats and short‐snouted taxa favoring swamps (Magnusson, 1989).

Crocodylian crowns are adapted for securing small prey whole and/or ripping off chunks of larger prey (Cleuren & De Vree, 2000; Grigg & Kirshner, 2015). There is typically no extensive processing, as these crowns are not well‐suited for cutting or shearing flesh into smaller sections (Drumheller et al., 2019; Fish et al., 2007). Combined with strong jaw muscles, they serve an opportunistic feeding strategy, primarily for aquatic and ambush predation (Drumheller et al., 2019; Horna et al., 2001; Hutton, 1987; Magnusson et al., 1987). All crocodylians employ a similar feeding mechanism involving grasping prey with the anterior end of the jaws and a sideways head motion. Smaller prey items may be shifted for a single killing bite, while hard‐bodied prey is positioned for effective crushing. With the largest prey, crocodylians may use the “death roll” behavior to dismember and swallow the prey (Drumheller et al., 2019; Fish et al., 2007).

Although crocodylians have been grouped based on snout characters (Brochu, 2001; Drumheller & Wilberg, 2020) and diet (Cleuren & De Vree, 2000; Laverty & Dobson, 2013; Magnusson et al., 1987) in several studies, quantitative dental morphology has rarely been used for this purpose. This study aimed to mathematically cluster modern crocodylian dentition based on morphology. Teeth from skeletal specimens of various species were sampled, and Euclidean measurements were taken, accounting for heterodonty. The results were linked to dietary and ecological information obtained from field observations and stomach content analysis, resulting in rigorous ecomorphological groups.

## 2 MATERIALS AND METHODS

### 2.1 Nomenclature

This study used the nomenclature in Smith and Dodson (2003) and expanded upon by Hendrickx et al. (2015). Mesial refers to the direction towards the symphysis of the jaw bone. Distal is the direction away from the jaw bone symphysis. Labial is the direction and surface of the crown facing outward, away from the tongue. Lingual is the crown surface and direction facing inward toward the tongue. Apical denotes the direction from the base of the crown to the apex of the crown. Basal denotes the direction from the apex to the base of the crown. Tooth position was indicated by either the presence of a tooth or an empty alveolus in the host bone. Teeth were lettered based on the host bone (premaxilla = P, maxilla = M, dentary = D), and numbered in ascending order from mesial to distal positions.

### 2.2 Specimens

Taxa chosen for this study were based on the variability of form, as well as accessibility, from the following collections: American Museum of Natural History, New York, NY (AMNH); Black Hills Institute of Geological Research, Hill City, SD (BHI); Field Museum of Natural History, Chicago, IL (FMNH); South Dakota School of Mines and Technology, Rapid City, SD (SDSM). Although these collections had the vast majority of species, the teeth often had fallen out of the skull. This limited the range of data that could be collected. Ultimately, data were collected from 48 specimens, spanning 18 of the 26 species currently recognized by the IUCN Crocodile Specialist Group, n.d. (Iucncsg.org—Crocodilian species) (Data S1). This includes members from all established ecomorphological categories based on snout shape (Brochu, 2001; Drumheller & Wilberg, 2020), and encompassed a wide range of sizes. Neonates were avoided. From these specimens, we collected data from 2330 individual teeth in total. Most teeth were in situ, but isolated teeth were also included if the specimen they belonged to and their position along the arcade was known.

### 2.3 Data collection

Smith et al. (2005) established the primary morphological methods used in this study, originally used to identify shed crowns from theropod dinosaurs (see also Hendrickx et al., 2014; Lubbe et al., 2009; Smith, 2005, 2007; Smith & Dalla Vecchia, 2006). Measurements were made using digital calipers. The base of the crown was defined by the point where the enamel ceases and it meets the root, which was often associated with the visible constriction of the tooth's length and width. Euclidean measures included the (1) Crown Base Length (CBL) or the maximum mesial‐distal distance of the crown [comparable to fore‐aft basal length used by previous authors (Currie et al., 1990; Larson & Currie, 2013; Torices et al., 2014)], (2) Crown Base Width (CBW) or the maximum labial‐lingual distance of the crown, (3) Apical Length (AL) or the distance from the most mesial point of the base to the apex, and (4) Crown Height (CH) or the distance from the distal‐most point of the base to the apex.

Only fully erupted teeth were included. Tooth quality was variable in extant specimens, and teeth with slightly worn apices were included. Teeth with very large facets or evidence of great postmortem damage were excluded. Cracks down the long axis of the teeth were common and were omitted if the crack distorted the shape of the tooth at the points where measurements were taken. Teeth on both sides of the mouth were measured, and, if both the left and right crowns were represented, the measurements were averaged together so as to not be overrepresented.

### 2.4 Size heterodonty normalizing

Size and shape heterodonty are decoupled in Crocodylia (D'Amore et al., 2019). Shape heterodonty is reflected by caniniform mesial teeth gradually transitioning to molariform distal ones, and size heterodonty is typically reflected by undulating patterns of large and small crowns. We attempted to factor out this size heterodonty in order to cluster specimens based on shape heterodonty alone. We therefore derived a size metric by calculating a simple volume for the tooth, and normalized Euclidean measurements based on this. This volume (V) was derived by modeling the tooth as an elliptical cone. The base of each tooth's volume was assigned as CBL and CBW. A measurement of tooth height (H) was also needed for conical volume, so CBL, AL, and CH were modeled as sides of a triangle and applied to Heron's formula (S), and from this area (A) was calculated:

$$S=\frac{\mathrm{CBL}+\mathrm{AL}+\mathrm{CH}}{2}$$

$$A=\sqrt{S*\left(S-\mathrm{CBL}\right)\times \left(S-\mathrm{AL}\right)\times \left(S-\mathrm{CH}\right)}$$

$$H=2\times \frac{A}{\mathrm{CBL}}$$

$$V=\frac{1}{3}\times \left[\pi \times \left(\frac{\mathrm{CBL}}{2}\right)\times \left(\frac{\mathrm{CBW}}{2}\right)\right]\times H$$

This volume is not intended to be an accurate value of the space the tooth occupies in reality, as the teeth may be foliate, curved, or keeled as opposed to perfectly conical. The advantage of this is that it is a consistent measurement that may be derived from all this data. This allows for the relative size of each Euclidean measurement to be considered without having to resort to proportions or arbitrarily selecting one raw measurement as a covariate. Normalizing a tooth dimension with the size of the tooth itself is also ideal for factoring out size heterodonty. If we instead used body size (such as the Head Width discussed below or a similar metric) as the covariate to derive residuals, all larger teeth would have positive residuals and smaller teeth would have negative ones, regardless of the shape of the tooth.

A series of reduced major axis regressions were then constructed, with the natural logarithm of the four Euclidean distance measures separately plotted against the natural logarithm of volume using PAST (Hammer et al., 2001). As Euclidean distances are one‐dimensional and volume is three‐dimensional, a slope of 1/3 was considered isometric. Residuals were derived from these regressions and used for subsequent analyses. This method not only reduced the effect of size heterodonty but also eliminated the need for calculating *z*‐scores.

### 2.5 Dividing the crocodylian tooth row

**FIGURE 1:**
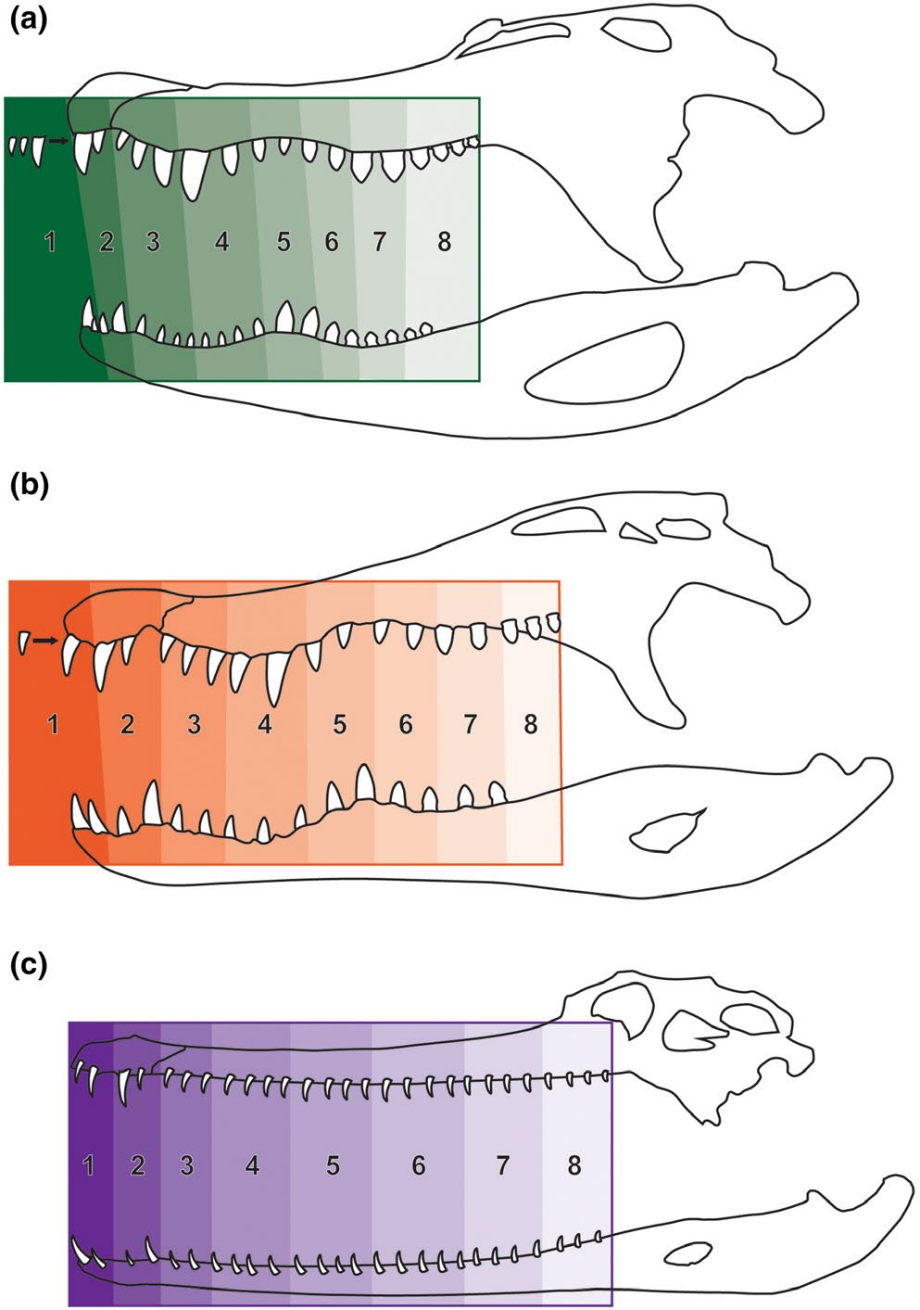
Diagrammatical representation of an alligatorid (a), crocodylid (b), and gavialid (c) with varied numbers of tooth positions, displaying how the arcade was divided up into eight sections. Note that any premaxillary teeth obscured from this perspective are depicted as separated from the jaw. Also, note that tooth P2 is missing from (b) and (c).

One issue that needed to be overcome was addressing variability in the number of teeth in the crocodylian arcade. Individual teeth could not be directly compared to one another by position, as the number of teeth along a tooth row varied greatly. For example, *Gavialis gangeticus* has almost twice as many tooth positions (54) as *Osteolaemus tetraspis* (31). Averaging all the teeth in the row together would obscure shape heterodonty, which we wanted to account for. Since tooth shape changes in a gradual, significantly linear fashion from mesial to distal (D'Amore et al., 2019), we assume that adjacent teeth are most similar to one another in shape. This therefore justifies breaking the dental arcade into sections, as each section would consist of teeth that did not differ greatly in said shape. Cranial and mandibular tooth rows were divided into eight sections of adjacent teeth (Figure 1), and measurements for all the teeth in each section were averaged. The number eight was entirely arbitrary; it was low enough for all sections to have at least one tooth even with teeth missing (which was frequent, see Data S1), yet high enough to account for the heterodonty as seen along the jaw. The result was a total of 32 values entered in the cluster analysis for each individual, with each being the averaged residuals of CBL, CBW, AL, and CH for all teeth in each of the eight jaw sections.

Dividing each individual's tooth row evenly by eight would have been consistent, but also would place functionally analogous teeth in different sections. We therefore structured our groups based on the regularly undulating pattern of enlarged versus smaller teeth along the arcades, ensuring that similar‐sized teeth were in the same groups across all species where present (Table 1). All crocodylians have an enlarged P4, D1, and D4, and most have enlarged M4/M5, M10/M11, and D10/D11 or D13/14 (Figure 1). Sections 1 and 2 are consistent between all species; with the enlarged D1 in section 1, and both P4 and D4 in section 2. Note that position P2 is commonly atrophied away (Brown et al., 2015; Webb & Messel, 1978), and was missing in section 1 in many specimens. Section 3 was entirely smaller teeth. Section 4 had the enlarged M4 in alligatorids or M5 in crocodylids/*Tomistoma schlegii*. Alligatorids differ from other crocodylians in that they have disproportionately more small dentary teeth midway along the arcade, so sections 3–4 had several more dentary crowns for this taxon. Section 5 had the enlarged distal dentary crowns, section 6 has only smaller crowns, and section 7 had the enlarged distal maxillary crowns. Section 8 varied considerably in size and number, but represented the distal‐most section of crowns. Most crocodylians have several maxillary teeth without corresponding dentary ones, due to the latter terminating several positions before the former. Section 8 therefore was often represented by more maxillary than dentary teeth. *G. gangeticus* and *T. schlegii* have clearly enlarged P4, D1, and D4, but also have many more distal positions with little to no size undulation. We therefore made sections 1–3 for *G. gangeticus* and 1–5 in *T. schlegii* consistent with the crocodylids, yet the remaining groups had several more positions added to each.

**TABLE 1:** Tooth positions that make up each dental category along the arcade.

Section	Crocodylidae		Alligatoridae		*Tomistoma*		*Gavialis*	
1	P1–3	D1–2	P1–3	D1–2	P1–3	D1–2	P1–3	D1–2
2	P4–5	D3–4	P4–5	D3–4	P4–5	D3–4	P4–5	D3–4
3	M1–3	D5–6	M1–3	D5–8	M1–3	D5–6	M1–3	D5–6
4	M4–5	D7–9	M4–5	D9–12	M4–5	D7–9	M4–7	D7–10
5	M6–7	D10–11	M6–7	D13–14	M6–7	D10–11	M8–11	D11–14
6	M8–9	D12–13	M8–9	D15–16	M8–10	D12–14	M12–15	D15–19
7	M10–11	D14–D15	M10–11	D17–18	M11–13	D15–D17	M16–19	D20–23
8	M12–14		M12–15	D19–22	M14–17	D18–19	M20–23	D24–26

In all specimens, only two were lacking any teeth in a single region. These were FMNH 69869 (*Paleosuchus palpebrosus*) and AMNH 137174 (*Paleosuchus trigonatus*), and both were lacking in section 2. Instead of eliminating these specimens entirely, we elected to extrapolate the values for section 2 by averaging the values for section 1 and section 3 when entering them into the cluster analysis.

### 2.6 Clustering crocodylians

Individual crocodylians were grouped mathematically using a k‐means cluster analysis in IBM SPSS Statistics v.30. Several methods were used to determine the appropriate number of clusters.

- An agglomeration schedule was produced by running a hierarchical cluster analysis using Ward's method (1963) in SPSS (Table S1). This produced coefficients that were plotted in a scree plot, and the “elbow rule” was then conducted. Five clusters appear to occupy the center of the “elbow” region of the plot best.
- Multiple k‐means cluster analyses were run with 2–7 clusters, and ANOVA tables were produced for each (see Table S2 for 5 clusters). Analyses with only 2 or 3 clusters yielded non‐significant ANOVA results for certain variables, suggesting that the optimal number of clusters is ≥4.
- The above multiple k‐means cluster analyses also yielded iteration histories, showing how many iterations it took to reach convergence. Fewer iterations indicate more stable clusters. Two and five clusters had the fewest iterations (three), four clusters had the most (seven), and the remainder all had four iterations.

Final cluster centers were graphed to determine how shape heterodonty was organized by cluster.

### 2.7 Head Width

Ontogenetic allometry strongly influences the feeding apparatus in crocodylians and has been linked with dietary shifts (Dodson, 1975; Drumheller & Wilberg, 2020; Erickson et al., 2003; Gignac & Erickson, 2015; Gignac & O'Brien, 2016; Hutton, 1987; Verdade, 2000; Webb & Messel, 1978; Wu et al., 2006). Although residual analyses were used to normalize for tooth size, the ontogenetic stage of the crocodylian specimen may play a role in where it ultimately clusters. Body size is frequently used as a measure of age and ontogeny, and we used the width of the head as our measure here. O'Brien et al. (2019) showed that head width accurately reflects both mass and length, and we follow a similar protocol. Head width was defined as the linear distance between the caudal‐most junction between the quadrate and quadratojugals on the lateral margins of the skull. Head width was then organized by cluster to determine if body size influences cluster assignment.

## 3 RESULTS

**FIGURE 2:**
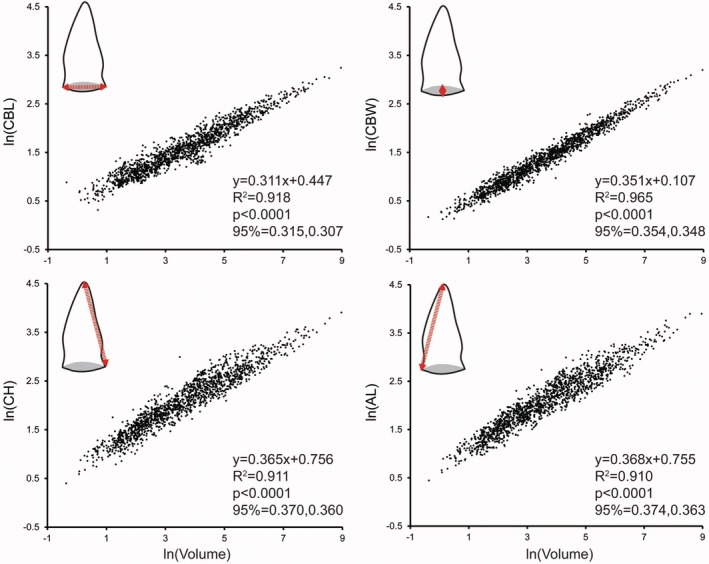
Natural logarithm (ln) of all Euclidean measurements of all crocodylian crowns, including crown base length (CBL), crown base width (CBW), crown height (CH), and apical length (AL) in mm, plotted against crown volume. Regression information for each bivariate plot is displayed, and residual values were derived from these.

Regression analyses indicated that Euclidean measures changed at similar rates as tooth size increased (Figure 2). All regressions were significant, with the goodness of fit values ≥91%. Both AL and CH regressions formed very similar slopes and intercepts when plotted against volume, which is indicative of the symmetrical nature of crocodylian teeth. Positive allometry (>1/3) showed that crocodylian teeth become relatively “tall” with increasing size. CBW also showed positive allometry, but CBL was negatively allometric. The y‐intercepts are particularly interesting, as CBL was greater than CBW's. This indicates that the regressions converge as crown size increases. Generally speaking, CBW tends to be less than CBL in smaller teeth, suggesting smaller crowns were more labiolingually compressed than larger ones, but the two are similar in relative size in larger teeth.

**FIGURE 3:**
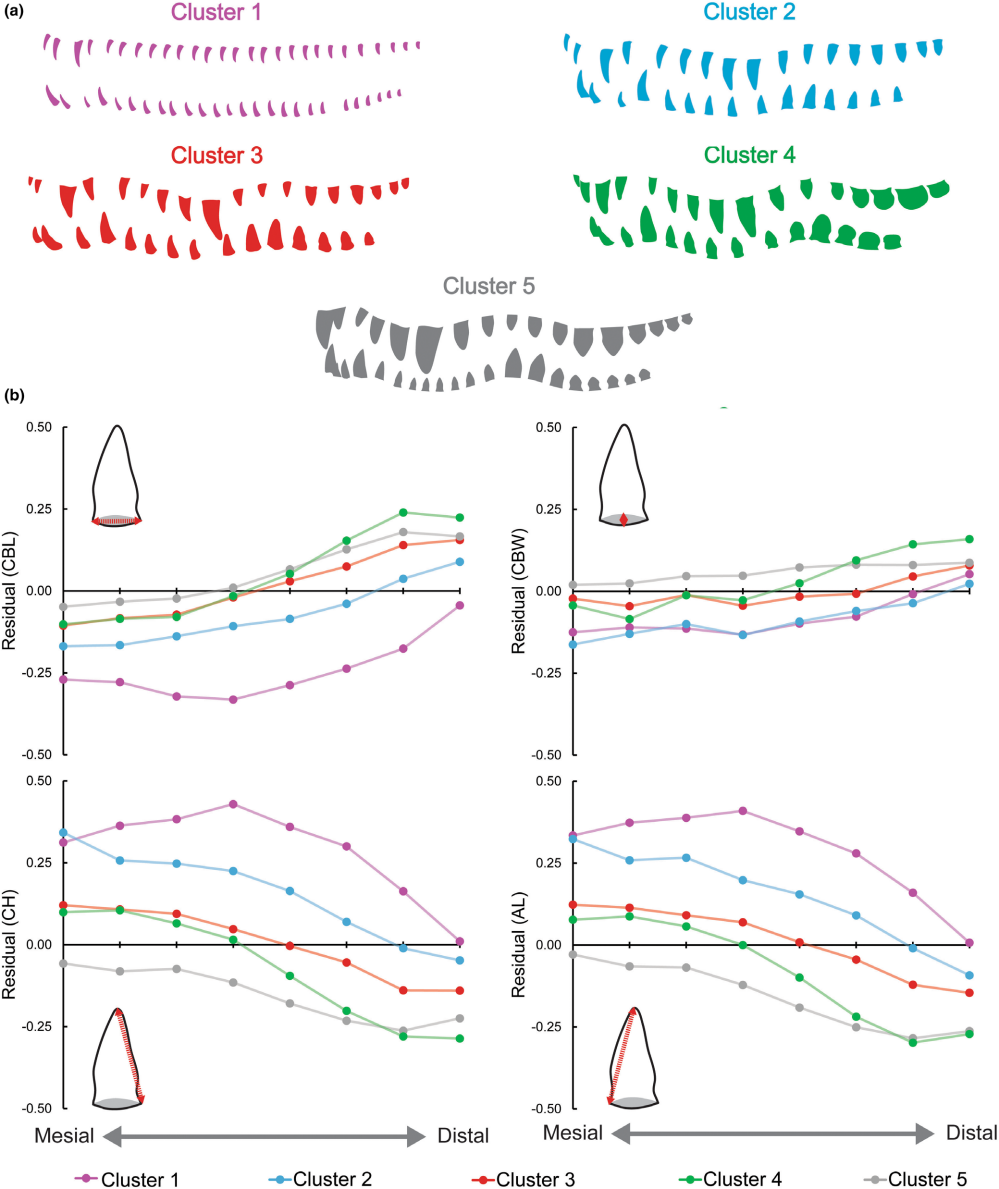
Diagrammatical representation of the typical dental morphotypes found in each cluster based on actual specimens (a). Cluster centers for crown base length (CBL), crown base width (CBW), crown height (CH), and apical length (AL) plotted for all eight sections of the arcade for all clusters (b).

Five clusters were determined to be optimal for clustering the crocodylian specimens (Figure 3). For all clusters, the final cluster centers of residual CBL and CBW increased from mesial to distal with few exceptions. This indicated that the crowns got relatively wider and longer at the base as one moves further back in the jaw. This change was typically more dramatic concerning CBL, with a greater change occurring from the most mesial to the most distal positions. This may be interpreted as distal teeth exhibiting a degree of labiolingual compression that mesial teeth lacked, although it is not seen in all clusters. Residual AL and CH showed the reverse trend, with a general decrease in the more distal positions. This indicates a general decrease in height from mesial to distal. AL and CH cluster centers are very similar for all groups at a given section and mirror one another along the arcade. This may be interpreted as representing teeth that did not have a mesial or distal lean to their apices.

Certain species have all representatives in one cluster, while others occupy two or three. The details of these clusters and their membership are as follows (Figure 3, Table 2):

1. Cluster 1 had the lowest residual CBL, and the highest AL/CH, in the final cluster centers, resulting in crowns that are tall, slender, uncompressed, and conical by a visible margin. They got proportionally taller towards the middle of the tooth row (section 4), after which they began to rapidly decrease in height towards the distal end. Unlike all other clusters indicating low labiolingual compression, Cluster 1 had CBW cluster centers that were always larger than their CBL counterparts. This cluster consisted solely of *G. gangeticus*.
2. Cluster 2 had low residual CBL/CBW and high AL/CH final cluster centers. These teeth may be described as tall and slender, but were not as extreme as those in Cluster 1. They also showed a degree of labiolingual compression that Cluster 1 lacked. All residuals showed gradual transitions from mesial to distal. It consisted of the smaller members of *P. triganotus*, and all *Mecistops cataphractus* and *T. schelgii* specimens.
3. Cluster 3's final centers rested between the others, and did not approach the upper or lower extremes of thickness or height. They showed a gradual transition from mesial to distal, increasing in length and thickness and decreasing in height. This cluster consisted of one *Alligator mississippiensis* specimen, several *Alligator sinensis, Caiman yacare, Crocodylus palustris*, and *Paleosuchus trigonous* specimens, and all *Caiman crocodilus, Crocodylus intermedius*, *Crocodylus siamensis, Melanosuchus niger*, *Osteolaemus osborni*, and *Paleosuchus palpebrosus* specimens.
4. The mesial crowns of Cluster 4 had very similar centers to Cluster 2, indicating that they were of moderate height and thickness. As one moved distal, the cluster centers approached and usually surpassed those of the most robust cluster (Cluster 5). These distal crowns were the squattest and least conical in shape. It consisted of one *A. sinensis*, *Ca. yacare, Cr. palustris*, and *Cr. porosus* specimen each, and all *Crocodylus acutus, Crocodylus rhombifer*, and *O. tetraspis* specimens.
5. Cluster 5 had the highest CBL and CBW, and the lowest AL and CH, final cluster centers for most of the arcade, resulting in consistently squat, conical crowns. This degree of “squatness” is only met/surpassed by the distal‐most sections of Cluster 5. All residuals showed relatively gradual transitions from mesial to distal. It consisted of some *Ca. yacare*, and *Crocodylus porosus*, most *A. mississippiensis*, and all *Cr. niloticus* specimens.

**TABLE 2:** Crocodylian species found within each cluster, and the number of individuals represented (in parentheses).

Cluster 1	Cluster 2	Cluster 3	Cluster 4	Cluster 5
*G. gangeticus* (2)	*A. mississippiensis* (1)	*A. mississippiensis* (4)	*T. schlegii* (2)	*A. sinensis* (1)
	*A. sinensis* (2)	Ca. *yacare* (1)	*M. cataphractus* (2)	Ca. *yacare* (1)
	Ca. *crocodilus* (4)	*Cr. intermedius* (1)	*P. trigonatus* (2)	*Cr. acutus* (2)
	Ca. *yacare* (3)	*Cr. niloticus* (3)		*Cr. palustris* (1)
	*Cr. palustris* (1)	*Cr. porosus* (1)		*Cr. porosus* (1)
	*Cr. siamensis* (2)			*Cr. rhombifer* (1)
	*M. niger* (2)			*O. tetraspis* (2)
	*O. osborni* (1)			
	*P. palpebrosus* (4)			
	*P. trigonatus* (1)			

*Note*: More details in the Data S1.

**FIGURE 4:**
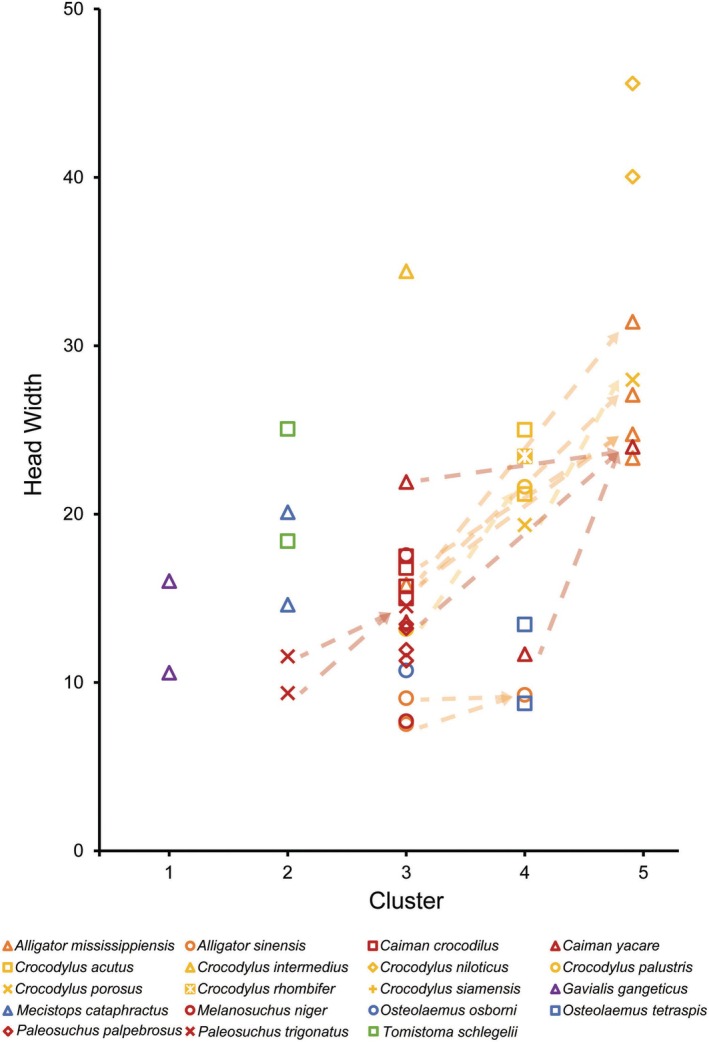
Head width (cm) plotted for all specimens organized by clusters. Arrows represent the movement of members of a single species from one cluster to another as they increase in size.

When body size was considered in the form of Head Width, the largest specimens were found in Cluster 5 (Figure 4). Almost all individuals with a width greater than 26 cm were in this cluster (except *Cr. intermedius*). In taxa that occurred in multiple groups including Cluster 5, the larger individual was always in Cluster 5. Many species were found in both Clusters 3 and 4, with *A. sinensis* and *Cr. palustris* having their largest individuals, and *Ca. yacare* having its smallest individual, in Cluster 4. The largest *P. triganotus* was found in Cluster 3, whereas the smaller ones were in Cluster 2.

## 4 DISCUSSION

Extant crocodylians were separated into five clusters based on dental morphology and position. As tooth size was normalized, the relative height (defined primarily by AL and CH) versus base dimensions (CBL and CBW) at a given position appeared to be the major factor dictating cluster assignment (Figure 3). Low height and high base measurements were indicative of crowns typically referred to as robust, and high heights and slender bases represented more gracile teeth. Variability in the degree to which teeth were robust/gracile along the dental arcade was indicative of the heterodonty inherent in the clade. The relative changes between CBL and CBW also indicated a degree of lateral compression, which was apparent in 4 out of 5 clusters in the distal portion of the arcade. Multiple families and subfamilies may be found in most clusters, and members of the same species were often found in more than one cluster. This indicates that cluster assignments were not entirely taxon specific, and were also influenced by size and ontogeny.

### 4.1 Influences on clusters

Cluster 1 may be described as having the most gracile teeth, as they were clearly the tallest relative to their base. Although often lumped together with other slender‐snouted taxa in the literature (Brochu, 2001; Drumheller & Wilberg, 2020), *G. gangeticus* was separated here. This separation may be further supported by unique skull morphospace occupation (Iijima, 2017; Pierce et al., 2008) and lower relative bite force output (Erickson et al., 2012). These crowns appear ideal for puncturing soft‐bodied prey with relatively little modification. The narrow base would result in low bending strength, but the unique lack of any lateral compression may be a mechanism to counter this slightly. Although not entirely piscivorous, most published research records a diet dominated by fish and small aquatic taxa (Saikia, 2012; Stevenson & Whitaker, 2010; Whitaker & Basu, 1982). The extreme crown height for the mesial and middle jaw regions of this taxon's substantial jaw length suggests that a singular function, prey acquisition, is prioritized. Because the middle of the jaw, specifically section 4, had the most gracile teeth, this may indicate it is better suited for prey capture. But Thorbjarnarson (1990) only found a minor difference in the percentage of fish captured with these two sections in *G. gangeticus* (44.8% for the mesial‐most third of the jaw, and 47.2% for the middle third), further supporting a consistency in function between these sections. Only the distal‐most crowns become robust. This may suggest this relatively small region is used for prey processing, although most accounts observe *G. gangeticus* swallowing prey whole (see Thorbjarnarson, 1990; Whitaker & Basu, 1982). These accounts do observe the repositioning of prey further back in the jaws during inertial feeding, and the reduced height of the distal teeth may make this process easier. Alternatively, the reduction in tooth height may simply be necessary for proper jaw closure (D'Amore et al., 2019).

Most other slender‐snouted taxa collectively fit into Cluster 2, whose teeth can be generalized into relatively tall and slender but not as extreme as Cluster 1. Both the teeth and snouts of these taxa are less resistant to mechanical stresses (Walmsley et al., 2013). Their diet consists mainly of prey smaller than they are in mass (Drumheller & Wilberg, 2020), including aquatic invertebrates and relatively small vertebrates (Grigg & Kirshner, 2015; Pauwels et al., 2003; Shirley, 2010; Tucker et al., 1996; Waitkuwait, 1989; Webb et al., 1982). Their teeth, coupled with their skull mechanics, seem ideal for smaller, compliant prey such as this; high strains associated with large, struggling prey, as well as the forces imposed by extensive prey processing, would more likely cause critical damage to the feeding apparatus. This is also a likely reason why these taxa tend to inhabit more open‐water and riverine habitats where their snouts are less likely to become entangled in the dense vegetation of debris (Borteiro et al., 2009; Magnusson et al., 1987). Members of Cluster 2 do more processing than Cluster 1, as the change to more robust crowns occurs gradually as opposed to abruptly. This implies a gradual increase in bending strengths with each region, allowing for the further breakdown of food as it is propelled toward the throat. This is supported by several accounts of *M. cataphractus* and *T. schlegii* eating larger and/or durable prey (Bezuijen et al., 1998; Brazaitis, 1973; Brown et al., 2021; Galdikas & Yeager, 1984; Groombridge, 1982; Selvaraj, 2012; Sideleau et al., 2022).

Cluster 3 has tooth dimensions that are situated between those of the more gracile Cluster 2 and the highly robust Cluster 5. There were numerous small‐ to medium‐sized taxa (Berkovitz & Shellis, 2017; Champagne et al., 2015; Grigg & Kirshner, 2015; Rivas et al.,^2023^; Velasco & Ayarzagüena, 2010), including members from all species of Caimaninae and *O. osborni*. The diets of these taxa are varied but tend to contain small prey items, including crustaceans, mollusks, reptiles, amphibians, rodents, and other small mammals, turtles, invertebrates, and a high percentage of fish (Berkovitz & Shellis, 2017; Borteiro et al., 2009; Brito et al., 2002; De Thoisy et al., 2006; Horna et al., 2001; Magnusson et al., 1987; Ősi & Barrett, 2011; Riley & Huchzermeyer, 2000; Santos et al., 1996).

Cluster 4 is unique in that it exhibits some of the greatest changes along the tooth row. This could display a higher degree of shape heterodonty, which itself may indicate a functional “division of labor” along the tooth row that is more pronounced than in most other clusters. Some of the smallest members of this cluster also have some of the most robust distal crowns, as expressed by the largest base and lowest height residuals (Eaton et al., 2009; Grigg & Kirshner, 2015; Jiang, 2010; Riley & Huchzermeyer, 2000). The distal teeth of *O. tetraspis* were some of the thickest and shortest teeth relatively speaking, giving them a bulbous, anvil‐like appearance. This correlates to a diet that includes a large number of hard‐shelled crustaceans and mollusks (Aoki, 1989; Berkovitz & Shellis, 2017; Brito et al., 2002; Diefenbach, 1979; Luiselli et al., 1999; McMahan et al., 2019; Pauwels et al., 2007; Riley & Huchzermeyer, 2000; Targarona et al., 2010; Waitkuwait, 1989). Several species of *Crocodylus* occur in this cluster that have not been recorded to have enlarged distal crowns like the species above, but they are still stout and robust enough in shape to place them in this cluster. This is appropriate as several are known to incorporate mollusks and turtles into their diet (Acosta‐Chaves et al., 2016; Thorbjarnarson, 1989).

Cluster 5 has the most robust crowns and was dominated by larger members of taxa typically referred to as generalists or macro‐generalists (Drumheller & Wilberg, 2020). These species capture prey up to or exceeding their body mass, respectively. Throughout their lives, they are known to incorporate a wide range of prey species, including invertebrates, fish, turtles, and large mammals (Daltry et al., 2003; Erickson et al., 2003; Fergusson, 2010; Hanson et al., 2015; Hutton, 1987; Whitaker & Whitaker, 1989; Whiting & Whiting, 2011). Their prey also increases in size and durability as they grow. For example, *A. mississippiensis* members greater than three meters in length had turtles as the most common reptile prey identified (Delany & Abercrombie, 1986; Rice et al., 2007). They are also known to consume vertebrate bones (Drumheller & Brochu, 2014, 2016; Njau & Blumenschine, 2006). Both behaviors align well with grown individuals needing crowns resistant to durable food items.

Of the species found in both Cluster 3 and 4, larger individuals were also typically found in Cluster 5 which was most likely due to the effects of ontogenetic allometry. This implies that throughout ontogeny teeth become more robust, as indicated by a relative decrease in height/increase in base size. Therefore, an individual may “graduate” into a more robust cluster after it reaches a certain size. This trend has been observed in *A. mississippiensis* (Erickson et al., 2003; Gignac & Erickson, 2015) and is believed to be an adaptation to niche shifts during ontogeny. Our data also suggests this trend may be expanded to include other taxa such as caimanines and crocodylids, and further supports the idea that the crocodylian feeding apparatus in general undergoes a noticeable transition with size increase and age. Iijima (2017) and D'Amore et al. (2019) both found an allometric increases in heterodonty throughout Crocodylia. Several studies have also found that snout shape changes ontogenetically in most species of crocodylians (Dodson, 1975), specifically in a u‐shaped progression resulting in significant snout widening (Iijima, 2017; Watanabe & Slice, 2014).

Slender‐snouted crocodylians do not appear to move into more robust categories, but this may or may not be an effect of our sample as ontogenetic skull widening has been observed in varying degrees in all these taxa (Iijima, 2017). Most slender‐snouted specimens are in Clusters 1 and 2, but *Cr. intermedius* is found in Cluster 4 (Brochu, 2001). As it is the largest representative of this morphotype, it may have “grown out” of Cluster 2 into the more robust Cluster 4. This hypothesis is supported by observed shifts in prey size, location, and durability frequently observed in slender‐snouted taxa (Bezuijen et al., 1998; Brazaitis, 1973; Brown et al., 2021; Galdikas & Yeager, 1984; Groombridge, 1982; Selvaraj, 2012; Sideleau et al., 2022). This may be explored further by incorporating a wider size range of slender‐snouted specimens. Future research should focus on the quantification of dentition in an ontogenetic series of individuals to rigorously determine how far‐reaching ontogenetic changes in dentition are in Crocodylia.

The fact that the smaller two *P. trigonatus* specimens fell in Cluster 2 makes for an interesting outlier, as they differ in numerous ways from the larger, slender‐snouted taxa they share a cluster with (Brochu, 2001; Drumheller & Wilberg, 2020). *P. trigonatus* teeth, superficially speaking, are clearly a different morphotype, so why did they end up clustered together? Upon closer inspection of the data, these *P. trigonatus* teeth overlapped these slender‐snouted taxa concerning all metrics except CBL (Data S1), so they apparently were of similar enough relative heights and thicknesses to cluster together. We believe this is due to a high degree of labiolingual compression without height reduction, giving the tooth a somewhat foliate appearance. Strong compression in small crocodylian taxa, and especially *Paleosuchus*, has been noted in several studies (Brochu, 2012; Cossette & Brochu, 2018). Compression and relative height also appear to decrease with ontogeny in many crocodylians, as stated above. It therefore stands to reason that the younger/smaller members of an already relatively compressed taxon would coincidentally share widths and heights (but not lengths) with more gracile‐toothed, slender‐snouted taxa. To further support this, the crowns of the largest *P. trigonatus* were noticeably shorter than its conspecifics. This, therefore, justified its position in a separate cluster.

### 4.2 Dentition across Crocodylia

In successfully clustering members of Crocodylia, several consistencies in the general nature of crocodylian dentition brought themselves to light. Prior work (D'Amore et al., 2019) had shown that shape heterodonty in Crocodylia was generally represented by relatively tall, caniniform, mesial teeth and squat, molariform, and distal teeth. The relative decrease in height, and the simultaneous increase in base length and thickness, along the arcade further supported these findings. This results in more molariform distal teeth, which have greater resistance to breaking than their mesial counterparts. Teeth closer to the hinge are subjected to greater pressures during a bite (Cleuren et al., 1995; Erickson et al., 2003, 2012, 2014; McHenry et al., 2006), and since most crocodylians incorporate some amount of hard prey into their diet (Brazaitis, 1973; Groombridge, 1982; McIlhenny, 1976; Nifong & Silliman, 2013; Ross & Magnusson, 1989; Santos et al., 1996; Selvaraj, 2012; Taylor, 1979) the distal teeth must be robust enough to hold and crush said prey. In addition, tall distal teeth would be ungainly, making it difficult to maneuver food for swallowing or even close the mouth. This is very much apparent in *G. gangeticus*, as the dramatic reduction in height in the distal‐most tooth positions may be a consequence of this necessity.

Dental occlusion plays a role in the number and position of teeth in crocodylians. Crocodylids and gavialids possess interdigitating teeth along much of their arcades (Brochu, 2001). Interdigitation implies there would be a similar number of teeth, and similar spacing between them, in the affected regions of both arcades. Due to their “overbite,” alligatorids lack interdigitation entirely. This allowed for different numbers, and consequently spacing, of teeth between the upper and lower arcades. Specifically, these species “crammed” numerous small teeth in the concavity midway along the dentary (Figure 1). One can speculate functional consequences, as numerous, closely spaced, similar‐sized teeth are better at gripping prey than puncturing (D'Amore et al., 2024; Mihalitsis & Bellwood, 2019). Prey positioned in this jaw region would be gripped by these dentary teeth while simultaneously punctured by the enlarged M4 on the opposite row. This may be particularly advantageous in securing large (Shoop & Ruckdeschel, 1990), slippery (Sampaio et al., 2013), and/or hard prey (Delany & Abercrombie, 1986; Drumheller & Brochu, 2014; Nifong & Silliman, 2013). It should also be noted that large, broad‐snouted crocodylids show a reduction in interdigitation, displaying a “semi‐overbite” condition as well as greater size heterodonty (Iijima, 2017). Although this does not alter overall tooth number, it may be functionally analogous to the alligatorid condition.

Lateral compression in modern Crocodylia is rarely mentioned in the literature (but see Brochu, 2012; Cossette & Brochu, 2018) yet was apparent to a degree in all taxa here except *G. gangeticus*. There are numerous possible explanations for this, and they may not be mutually exclusive. Compression may be an adaptation for food processing. Compressed distal crowns could sheer more compliant food items, so species that focus on fish, small vertebrates, and/or arthropods may more effectively process prey. Larger generalist and macro‐generalist crocodylians break down more vertebrate bones (Drumheller & Brochu, 2014; Njau & Blumenschine, 2006). That, coupled with allometrically greater bite forces (Erickson et al., 2012, 2014), would provide the selection pressure for the decrease in compression in larger individuals as seen here. Compression, or the lack thereof, may also be a functional constraint of the jaw mechanism. Monfroy (2017) showed these compressed teeth have greater bending strength in the mesial‐distal direction. Based on their position, the mesio‐distal axis of these teeth rotates about the hinge during jaw closure. As this axis is wider, it would have greater bending strength during jaw closure, and resist the forces imposed on the teeth by a food item better. These forces may be compounded by the fact that struggling prey would most likely resist along this axis as well (Monfroy, 2017). The fact that the mesial‐most teeth in Crocodylia display the least compression further supports this functional constraint hypothesis. Due to the rounding of the rostrum these teeth would instead rotate about their labiolingual axis, an axis which compression would only weaken. This trend of labiolingual expansion in the mesial‐most crowns may be commonality in sauropsids, exhibited by relatively lower compression in the premaxillary crowns of ziphodont varanids (Maho & Reisz, 2024; D. D'Amore personal observation) and the propensity for the “D‐shaped” morphotype in theropods (Carr & Williamson, 2004; Currie et al., 1990; D'Amore, 2009; Hendrickx et al., 2015; Molnar, 1978; Smith, 2005).

Regardless of its utility, lateral compression in modern crocodylians is slight relative to their extinct ziphodont relatives (Larsson & Sues, 2007; Ristevski et al., 2024; Sobbe, 2013). This may be a consequence of the methods typically used by modern taxa to disarticulate/dismember prey. The death roll is employed by many crocodylians during feeding, whereby the crocodylian grips the prey with the mesial crowns and rotates its body rapidly (Fish et al., 2007; Grigg & Kirshner, 2015). Many crocodylian taxa consume large mammalian prey that must be dismembered in order to be swallowed, and it was previously believed these taxa were more apt to employ the death roll (Daltry et al., 2003; Erickson et al., 2003; Fergusson, 2010; Hutton, 1987; Whitaker & Whitaker, 1989). However, experimental research has shown the vast majority of crocodylian species death roll for feeding and/or defense (Drumheller et al., 2019). This rolling motion would theoretically incur great stresses on the teeth, especially about the labiolingual axis. The degree of lateral compression seen in ziphodont archosaurs could cause the tooth to fail under such stresses, and strong compression may have been selected against as a result of rolling behaviors.

### 4.3 Limitations and future work

This was not an exhaustive study of all modern crocodylian species or sizes, mainly for logistical reasons such as teeth falling out of the available skulls. Therefore, as several species could not be included, notably some with dentition described as very gracile (*Crocodylus johnstoni*) or very robust (*Caiman latirostris*). Additionally, modern crocodylians are notorious for being taxonomically misidentified, with groups traditionally referred to as a single species or subspecies complexes broken up on numerous occasions. Examples included *Crocodylus suchus* splitting from *Cr. niloticus*, and *Mecistops leptorhynchus* splitting from *M. cataphractus*, and the number of species within *Osteolaemus* (Eaton et al., 2009; Hekkala et al., 2011; Shirley et al., 2018). These newer species designations are often not yet accounted for in museum collections, or have not been properly reassigned yet. Future research should consider dry specimens from other collections or doing a similar study using CT scans of more species with more intact teeth.

Concerning *Osteolaemus*, our data suggests that tooth shape may be a potential method for distinguishing between species. An interesting observation was the degree of variation between our *O. osborni* and *O. tetraspis* specimens; with the former lacking the extreme distal teeth of the latter and segregating them to different clusters. Future research may focus on determining if certain traits outlined here may potentially distinguish closely related taxa from one another.

Averaging all the teeth in the eight tooth row sections was advantageous in that it allowed us to compare species with vastly different numbers of tooth positions. An inherent consequence of this is that different numbers of tooth positions were represented in certain sections based on the taxon, adding a degree of subjectivity to this method. This was necessary though, and elucidates the anatomical differences in the feeding apparatuses of these different taxa. As discussed above alligatorids had dentary teeth overrepresented in sections 3 and 4. Dentary teeth had a variable degree of inclusion in Section 8, and in the case of crocodylids none at all. Last, *G. gangeticus* and *T. schlegii*, being the only “actual” longirostrine taxa (according to Brochu, 2001, p. 572), had longer snouts and more tooth positions than the other interdigitating taxa. This almost certainly functioned to obscure the dramatic degree of morphological change that is apparent in the distal‐most crowns, especially in *G. gangeticus*. We assert that, because of the linear nature of shape change along the tooth row (D'Amore et al., 2019), this most likely would not affect cluster assignments. Nevertheless, future studies may consider this in heterodonty work.

We did not take into account rearing conditions for the specimens in our study and little work has been done to see if these influence tooth shape. Skull shape and tooth orientation may be irregularly influenced by captive rearing (Drumheller et al., 2016; Erickson et al., 2004), but D'Amore et al. (2019) found that wild‐caught individuals and those designated as “no data” had no significant differences in tooth morphology. Future studies should explore this further to see if rearing conditions play a major role in tooth development.

Because certain morphological and biomechanical consistencies in dentition exist between crocodylian taxa, ecomorphological categories may be constructed. These can take into account the data presented here and place them in the context of body size and published accounts of prey consumed. These groups should be understood in the context that all crocodylians are fundamentally opportunistic predators, and we by no means suggest that potential prey items would be “off the menu” for most taxa. If we understand the relationship between skull morphology, crown morphology, and prey preference/ecological niche in extant taxa, we can make comparisons to fossil taxa with similar morphologies through analogy (Ciampaglio et al., 2005; Massare, 1987). The knowledge gained from extant crocodylian taxa here can be applied to better understand the ecology of their fossil relatives. By collecting the measurements on fossil crowns, we can make a better approximation of their position in the ecosystem. Much emphasis is placed on crown morphology in fossil crocodilians when hypothesizing their role in prehistoric ecosystems, and these techniques may be used to directly compare modern taxa with fossils. Even fossil crocodylians considered to be “heterodont” (Ősi, 2014) or ziphodont (Larsson & Sues, 2007; Ristevski et al., 2024) may still be evaluated using these methods. The alligatoroid *Brachychampsa* has abnormally squat, rounded‐off distal crowns believed to be ideal for a durophagous diet of turtles and bivalves (Carpenter & Lindsey, 1980; D'Amore et al., 2019; Norell et al., 1994; Sullivan & Lucas, 2003). We suspect the crowns of *Brachychampsa* may place it in its own unique cluster, along with other strongly globidont taxa.

It is our hope that future researchers will use the database of Euclidean measurements accumulated here. This may be added to with new extant or fossil crocodylians to bolster the sample size and range of species represented. It may also be used for direct comparisons with other taxa, as these measurements are often taken in non‐mammalian tetrapods. A massive database of theropod dinosaur teeth has been using similar measurements (Hendrickx et al., 2020 and references within), and combining the two will hopefully inspire future meta‐analyses of broader archosaur dentition.

## 5 CONCLUSIONS

Crocodylia was successfully separated into five clusters based on Euclidean dental morphology. Using cluster analysis contributes a degree of rigor to the process of separating taxa based on morphology, as the number and thresholds of the resultant morphological groups are mathematically dictated. Accounting for heterodonty by breaking up the jaw into sections allowed for a more detailed look at dental variability between individuals, which may not have been possible if a cherry‐picked tooth or the average of all teeth had been used. This is not without subjectivity though, as the method we used to section the tooth rows was arbitrarily based on the number of positions and intact teeth. Nevertheless, this is one of the few studies to separate modern crocodylians into ecomorphological groups using non‐qualitative measurements of the dentition.

The nature of heterodonty appears to be influenced by the differences in the direction and degree of forces applied to a given tooth. This is primarily based on its position and the mechanical properties of the diet of the cluster members, particularly how large and durable major prey items are. The clusters vary by the degree to which the teeth are gracile or robust, rounded or compressed, and how much these characters change from mesial to distal. This correlates broadly to both snout shape, which is influenced by similar selection pressures concerning feeding. Cluster membership also may be explained by ontogenetic changes, as we draw a tentative correlation between size and dental robustness in species that occupy more than one cluster.

## AUTHOR CONTRIBUTIONS

Jason Testin contributed to this work by proposing the original research question of exploring the relationship between tooth morphology in extant crocodylians and prey preference. He also did the majority of the literature reviews to summarize the published data on stomach content and field observations of prey. Domenic D'Amore contributed to the work by proposing the method and carrying out the statistical analysis of the collected morphological data. He was responsible for identifying and describing the characteristics of the clusters that resulted from the analysis. Both Mr. Testin and Dr. D'Amore collected morphological data measurements from crocodylian material in museum collections for the construction of the database of samples.

## CONFLICT OF INTEREST STATEMENT

The authors declare no conflict of interest.

## Supporting information


Tables S1–S2.

Data S1.

## Data Availability

The data supporting the findings of this study are available within the supporting documents to this article. For additional information, please contact the corresponding author.
