# Supplementary material for: Clustering crocodylian dental morphology: Insights into functional adaptations, diet, and ontogeny
Source: J Anat. 2025 Jul 2;248(1):1–17. doi: 10.1111/joa.70014 (PMC12682598; doi:10.1111/joa.70014)
Supplement: Supplementary file 1 — Tables S1–S2. [file JOA-248-1-s002.docx]

**Table S1**: Agglomeration schedule to determine cluster number

| **Stage** | **Cluster Combined** | | **Coefficients** | **Stage Cluster First Appears** | | **Next Stage** |
| --- | --- | --- | --- | --- | --- | --- |
|  | **Cluster 1** | **Cluster 2** |  | **Cluster 1** | **Cluster 2** |  |
| 1 | 42 | 43 | 0.021 | 0 | 0 | 7 |
| 2 | 18 | 19 | 0.045 | 0 | 0 | 14 |
| 3 | 12 | 16 | 0.074 | 0 | 0 | 16 |
| 4 | 14 | 27 | 0.114 | 0 | 0 | 21 |
| 5 | 10 | 38 | 0.155 | 0 | 0 | 29 |
| 6 | 4 | 5 | 0.197 | 0 | 0 | 38 |
| 7 | 37 | 42 | 0.240 | 0 | 1 | 11 |
| 8 | 39 | 44 | 0.288 | 0 | 0 | 30 |
| 9 | 23 | 26 | 0.337 | 0 | 0 | 12 |
| 10 | 40 | 41 | 0.386 | 0 | 0 | 42 |
| 11 | 37 | 47 | 0.438 | 7 | 0 | 29 |
| 12 | 1 | 23 | 0.493 | 0 | 9 | 23 |
| 13 | 25 | 29 | 0.550 | 0 | 0 | 19 |
| 14 | 18 | 20 | 0.608 | 2 | 0 | 28 |
| 15 | 46 | 48 | 0.667 | 0 | 0 | 39 |
| 16 | 12 | 15 | 0.730 | 3 | 0 | 22 |
| 17 | 6 | 13 | 0.797 | 0 | 0 | 33 |
| 18 | 11 | 45 | 0.867 | 0 | 0 | 30 |
| 19 | 7 | 25 | 0.944 | 0 | 13 | 33 |
| 20 | 33 | 34 | 1.030 | 0 | 0 | 27 |
| 21 | 14 | 28 | 1.120 | 4 | 0 | 28 |
| 22 | 9 | 12 | 1.211 | 0 | 16 | 32 |
| 23 | 1 | 22 | 1.311 | 12 | 0 | 34 |
| 24 | 17 | 21 | 1.417 | 0 | 0 | 31 |
| 25 | 35 | 36 | 1.525 | 0 | 0 | 27 |
| 26 | 2 | 8 | 1.640 | 0 | 0 | 32 |
| 27 | 33 | 35 | 1.754 | 20 | 25 | 43 |
| 28 | 14 | 18 | 1.877 | 21 | 14 | 36 |
| 29 | 10 | 37 | 2.003 | 5 | 11 | 37 |
| 30 | 11 | 39 | 2.130 | 18 | 8 | 40 |
| 31 | 17 | 24 | 2.269 | 24 | 0 | 34 |
| 32 | 2 | 9 | 2.408 | 26 | 22 | 40 |
| 33 | 6 | 7 | 2.550 | 17 | 19 | 36 |
| 34 | 1 | 17 | 2.707 | 23 | 31 | 35 |
| 35 | 1 | 3 | 2.924 | 34 | 0 | 38 |
| 36 | 6 | 14 | 3.144 | 33 | 28 | 44 |
| 37 | 10 | 30 | 3.373 | 29 | 0 | 39 |
| 38 | 1 | 4 | 3.606 | 35 | 6 | 42 |
| 39 | 10 | 46 | 3.843 | 37 | 15 | 45 |
| 40 | 2 | 11 | 4.107 | 32 | 30 | 44 |
| 41 | 31 | 32 | 4.391 | 0 | 0 | 43 |
| 42 | 1 | 40 | 5.004 | 38 | 10 | 46 |
| 43 | 31 | 33 | 5.623 | 41 | 27 | 47 |
| 44 | 2 | 6 | 6.363 | 40 | 36 | 45 |
| 45 | 2 | 10 | 7.707 | 44 | 39 | 46 |
| 46 | 1 | 2 | 11.401 | 42 | 45 | 47 |
| 47 | 1 | 31 | 18.647 | 46 | 43 | 0 |

**Table S2**: Analysis of variance (ANOVA) table for variable within five clusters.

| **Variable** | **Cluster** | | **Error** | | **F** | **P** |
| --- | --- | --- | --- | --- | --- | --- |
|  | **Mean^2^** | **df** | **Mean^2^** | **df** |  |  |
| CBL1 | 0.027 | 4 | 0.003 | 43 | 8.549 | <0.001 |
| CBL2 | 0.034 | 4 | 0.003 | 43 | 11.115 | <0.001 |
| CBL3 | 0.042 | 4 | 0.005 | 43 | 8.899 | <0.001 |
| CBL4 | 0.059 | 4 | 0.003 | 43 | 17.798 | <0.001 |
| CBL5 | 0.070 | 4 | 0.004 | 43 | 16.252 | <0.001 |
| CBL6 | 0.090 | 4 | 0.004 | 43 | 21.713 | <0.001 |
| CBL7 | 0.090 | 4 | 0.005 | 43 | 18.257 | <0.001 |
| CBL8 | 0.037 | 4 | 0.005 | 43 | 6.787 | <0.001 |
| CBW1 | 0.036 | 4 | 0.006 | 43 | 6.459 | <0.001 |
| CBW2 | 0.026 | 4 | 0.004 | 43 | 6.022 | 0.001 |
| CBW3 | 0.024 | 4 | 0.006 | 43 | 4.258 | 0.005 |
| CBW4 | 0.035 | 4 | 0.004 | 43 | 8.344 | <0.001 |
| CBW5 | 0.032 | 4 | 0.004 | 43 | 7.947 | <0.001 |
| CBW6 | 0.039 | 4 | 0.004 | 43 | 10.380 | <0.001 |
| CBW7 | 0.035 | 4 | 0.004 | 43 | 8.489 | <0.001 |
| CBW8 | 0.020 | 4 | 0.004 | 43 | 4.562 | 0.004 |
| CH1 | 0.164 | 4 | 0.005 | 43 | 36.170 | <0.001 |
| CH2 | 0.142 | 4 | 0.003 | 43 | 43.514 | <0.001 |
| CH3 | 0.142 | 4 | 0.004 | 43 | 37.319 | <0.001 |
| CH4 | 0.181 | 4 | 0.004 | 43 | 51.215 | <0.001 |
| CH5 | 0.193 | 4 | 0.005 | 43 | 36.555 | <0.001 |
| CH | 0.196 | 4 | 0.004 | 43 | 50.424 | <0.001 |
| CH7 | 0.141 | 4 | 0.004 | 43 | 38.196 | <0.001 |
| CH8 | 0.080 | 4 | 0.005 | 43 | 17.533 | <0.001 |
| AL1 | 0.139 | 4 | 0.006 | 43 | 22.895 | <0.001 |
| AL2 | 0.141 | 4 | 0.004 | 43 | 38.350 | <0.001 |
| AL3 | 0.150 | 4 | 0.004 | 43 | 40.888 | <0.001 |
| AL4 | 0.171 | 4 | 0.004 | 43 | 41.701 | <0.001 |
| AL5 | 0.196 | 4 | 0.004 | 43 | 52.231 | <0.001 |
| AL6 | 0.224 | 4 | 0.004 | 43 | 55.547 | <0.001 |
| AL7 | 0.170 | 4 | 0.003 | 43 | 51.507 | <0.001 |
| AL8 | 0.069 | 4 | 0.006 | 43 | 11.110 | <0.001 |
